# Status Asthmaticus in the Pediatric ICU: A Comprehensive Review of Management and Challenges

**DOI:** 10.3390/pediatric16030054

**Published:** 2024-07-31

**Authors:** Amy Joseph, Hammad Ganatra

**Affiliations:** Pediatric Critical Care Medicine, Pediatric Institute, Cleveland Clinic, 9500 Euclid Ave, Cleveland, OH 44195, USA; josepha11@ccf.org

**Keywords:** asthma, status asthmaticus, PICU asthma, management of asthma

## Abstract

This narrative review addresses the significant burden of pediatric status asthmaticus, which comprises almost 20% of admissions to pediatric intensive care units (PICUs). It highlights the diverse modalities employed in the PICU for managing this life-threatening condition, and thoroughly discusses the literature in support of or against these treatment modalities.

## 1. Introduction

Asthma is an obstructive airway disorder secondary to airway hyperresponsiveness, bronchospasm, airway inflammation, mucosal edema, and mucus plugging. Status asthmaticus, also known as acute severe asthma, is characterized by an asthma attack that is unresponsive to repeated doses of inhaled β-agonists, oral or IV steroids, and oxygen therapy, requiring hospital admission [1,2]. Patients with status asthmaticus typically present with wheezing, respiratory distress, and cough and are oftentimes associated with a viral or allergen trigger. Allergen triggers can often include pollen, and, as such, it is important to understand the native plant and tree pollen. Avoidance of said triggers is crucial to preventing acute asthma exacerbation [3]. Examination findings often include wheezing, hypoxemia, and signs of respiratory distress such as tachypnea and accessory muscle use. Acute severe asthma can present with a prolonged expiratory phase and biphasic, or both inspiratory and expiratory, wheezing. An unfavorable sign of status asthmaticus is a “silent chest”, which can be indicative of a complete lack of airflow and may signal impending respiratory failure [2].

While status asthmaticus is primarily a clinical diagnosis, laboratory and imaging findings can be supportive evidence for the diagnosis. In early stages of status asthmaticus, arterial blood gas analysis can show hypoxemia with hypercapnia. In later stages, this may then transition to hypoxemia with normal P_a_CO_2_, albeit in the setting of signs of significant respiratory distress. Blood chemistry analysis may show signs of dehydration, and complete blood cells could show signs of leukocytosis, though neither are pathognomonic for acute severe asthma. A chest X-ray when obtained oftentimes shows signs of hyperinflation and can also reveal other clinically relevant conditions, such as pneumothorax, pneumonia, atelectasis, or other conditions that may be masquerading as asthma, such as a foreign body aspiration or congenital airway anomalies [1].

Various scores have been utilized for grading the severity of asthma, and thereby using them for determining admission to the pediatric intensive care unit (PICU) and management stratification. Examples of the most commonly used asthma scoring tools are summarized in Table 1.

Status asthmaticus is a clinical emergency, and understanding of various management strategies available to clinicians is imperative. A recent literature review has not been published, and we look to erase that deficit with this narrative review.

## 2. Epidemiology

Asthma is the most common chronic disease in the pediatric population, and its prevalence has continued to increase. About 10% of all children in the United States (US) have been diagnosed with asthma [7] and account for about 20% of all admissions to the pediatric intensive care unit (PICU). Pediatric mortality due to asthma in the United States accounts for about 10.6 deaths per million. Among pediatric deaths due to asthma, males and non-Hispanic black children make up the majority [8].

Pediatric asthma also poses a financial burden on the US healthcare system and on the individual patient and family. The average cost per child with asthma per year in the US in 2017 ranged to USD 3076, an annual cost that is significantly higher than for children without asthma. The national financial direct cost of pediatric asthma was estimated to be about USD 5.92 billion, with acute care visits accounting for a significant portion of those costs. Overall, pediatric asthma imposes considerable financial strain [9].

## 3. Medical Management

Medical management of status asthmaticus in the pediatric ICU setting is multimodal, and a good understanding of different therapies is imperative to appropriately treat these patients. There is no single best standard practice for managing status asthmaticus; rather, there is a series of clinical care options that must each be carefully considered and navigated based on the specific severity and circumstances of each patient. In this section, we will discuss the various aspects of medical management.

**β-agonists**

β-agonists are the first line of treatment of status asthmaticus. Typical β-agonist agents include albuterol, epinephrine, terbutaline, and isoproterenol. β-adrenergic receptors are present on airway smooth muscle cells, particularly β_2_ receptors. Stimulation of these receptors results in stimulation of adenylyl cyclase and causes increased intracellular cAMP and thereby enhanced activity of cAMP-dependent protein kinase A, which will then cause airway relaxation and inhibits release of mast cell mediators that can cause bronchoconstriction. β receptor agonists for treatment of status asthmaticus are best delivered by inhalation so as to have a maximal local airway smooth muscle effect and the least systemic toxicity. Most of the side effects from beta-receptor agonists occur from activation of β_1_ receptor agonism, such as tachycardia, tremors, nausea, and decrease in extracellular potassium levels [10]. Beta receptor agonists can also result in type B lactic acidosis due to overstimulation of the beta receptors [11].

**Albuterol**

Albuterol is a β_2_ selective agonist and it is the most commonly used adrenergic agonist that is used for management of status asthmaticus. In the US, albuterol is available via inhalation, either as a metered dose inhaler or a nebulized treatment. While oral formulations of albuterol are also available, they are not recommended over aerosol therapy due to slower onset of action and more adverse effects [12]. Intravenous albuterol (salbutamol) is available outside of the US, though its efficacy seems to have mixed results in different studies. Intravenous routes have not been proven to have superior results in comparison to nebulized albuterol, and there have been increased side effects of hypokalemia and tachycardia noted [13,14]. Intravenous salbutamol has been advocated for use outside of the US for patients who are responding to nebulized albuterol poorly, those who are coughing significantly, or are in extremis despite inhalational therapy [14]. In this section, we will be primarily addressing nebulized albuterol given that it is the most common form of albuterol used in status asthmaticus in the US.

Albuterol is a mixture of R-albuterol (racemic albuterol) and S-albuterol. R-albuterol is the active enantiomer that causes bronchodilation and S-albuterol is thought to be inactive in humans. Pure R-albuterol is available in the form of levalbuterol. The purported benefit of levalbuterol is that there are decreased adverse effects in comparison to racemic albuterol. However, evidence does not suggest that using levalbuterol is superior to R-albuterol in terms of clinical efficacy, nor does it have decreased adverse effects, including changes in heart rate [15].

During the initial treatment phase of asthma, albuterol nebulizer is typically administered at a dose of 0.05–0.15 mg/kg every 20 min. For patients that are not able to remain without an albuterol dose for more than 1 h, then continuous albuterol should be given. In children with severe asthma exacerbation, continuous albuterol has shown a significantly higher rate of treatment success and lower requirement to escalate to more aggressive therapies in comparison to intermittent nebulization [16]. Continuous albuterol is usually dosed at 0.15–0.5 mg/kg/h, to a maximum of 10–20 mg/h [1,2,17]. Higher doses have not been shown to be more effective or to decrease hospital length of stay, though there is a higher incidence of adverse effects, including hemodynamic effects such as tachycardia, lower mean arterial pressure, and lower diastolic blood pressure [1]. Albuterol nebulizers can be administered via various interfaces, including face masks, high-flow nasal cannula, BiPAP/C-PAP, or endotracheal tube, and can thus be used in conjunction with respiratory devices that can offer more respiratory support.

**Epinephrine**

Epinephrine is a nonselective adrenoreceptor that can result in rapid bronchodilation. It is typically given either subcutaneously, intravenously, intramuscularly, or is inhaled. Epinephrine stimulates α, β_1_, and β_2_ receptors and can thus result in tachycardia and arrhythmias as well. An advantage of epinephrine is the ability to utilize it without IV access and it can also be beneficial in patients with limited airflow resulting in decreased distribution of inhaled agents. It is not used as often now, given the availability of more selective β receptor subcutaneous or IV drugs [10], but it maintains its utility in emergent situations where IV access has not been established yet. Epinephrine dosing used in these cases is 0.01 mg/kg/dose to a maximum of 0.5 mg and it can be given every 20 min up to 3 doses [14].

**Terbutaline**

Terbutaline is another β_2_ selective agonist that can be used for status asthmaticus management. Terbutaline is available as a metered dose inhaler (outside of the US), oral tablet, subcutaneous injection, or intravenously. The oral form of terbutaline is not used often, given that it has a slow onset of action and can have more adverse effects [10]. The subcutaneous route can be advantageous in patients with limited airflow and no IV access. Dosage of terbutaline given subcutaneously is typically 0.1 mg/kg/dose with a maximum of 0.3 mg, and it can be repeated every 15–20 min for three doses if needed. If being given IV, it is usually given with a loading dose of 10 mcg/kg and followed by a continuous infusion at 0.1–10 mcg/kg/min. The continuous infusion is often started low, 0.1–0.5 mcg/kg/min, based on clinical practice, and titrated up for clinical effect or until adverse effects arise [2]. Side effects that can be seen due to terbutaline are tremors, skeletal muscle twitches, hypokalemia, tachycardia, and hypotension (particularly diastolic hypotension). There are rare instances of increased cardiac enzymes measured in patients on continuous terbutaline infusion, though this was not associated with cardiac dysfunction. Very rarely, there have been reports of ventricular tachyarrhythmias while on continuous IV terbutaline that would resolve with a decrease in dosing. It is recommended that patients on IV terbutaline remain on continuous telemetry to monitor for tachyarrhythmias. IV terbutaline has not been found to be more efficacious in treating severe acute asthma than other IV medications used for this reason such as IV methylxanthines or IV aminophylline [2,13,18].

**Long-Acting Beta Agonists**

Long-acting β agonists are part of the mainstay of management as a controller medication for asthma. However, there is no published literature to establish their role in the management of status asthmaticus. Following resolution of status asthmaticus, regular usage of a controller medication is imperative in preventing acute asthma exacerbation [2].

**Steroids**

Corticosteroids are important in the management of status asthmaticus given that the primary driver of the exacerbation is inflammation [19]. Glucocorticoids decrease airway inflammation via suppression of a multitude of cytokines, adhesion molecules, and inducible enzymes (nitric oxide synthase and cyclooxygenase 2). They also cause upregulation of certain genes, including β_2_ receptor and lipocortin-1, and decrease airway mucus production and capillary permeability [1,2].

Glucocorticoids given for acute severe asthma should either be given oral or IV and should be given within 1 h of presentation. There is no proven benefit of inhaled corticosteroids in status asthmaticus. The oral form most used is prednisone or prednisolone. IV methylprednisolone is the most common form used parenterally, but dexamethasone and hydrocortisone can also be used. IV forms should be used in patients unable to take steroids enterally due to nausea and vomiting or NPO due to respiratory failure. Oral prednisone and IV methylprednisolone are equally efficacious in treating status asthmaticus with no differences in rates of hospital admission when used in the emergency department or hospital length of stay, though parenteral route should be used for the reason discussed above. Oral prednisone proves to be a more cost-effective option compared to IV methylprednisolone [20,21]. Oral prednisolone is administered at 1–2 mg/kg/day, given 1–2 times per day [22].

Studies looking at IV methylprednisolone, IV dexamethasone, and IV hydrocortisone have shown that length of hospital stay and continuous β_2_ agonist therapy is comparable with all three medications for status asthmaticus in the PICU. Each has similar safety profiles, though use of hydrocortisone was associated with higher mean arterial pressures in comparison to the others. Typical dosing regimens that were used in these studies were IV methylprednisolone 0.5–1 mg/kg/dose every 6 h with a maximum of 60 mg/dose for 5 days or based on clinical improvement, IV hydrocortisone 1.5–4 mg/kg/dose every 6–8 h until discontinuation of continuous albuterol nebulization, and IV dexamethasone 0.6–1 mg/kg/day divided every 6 h for a total of 2–3 days [23,24]. Oftentimes, the choice of which IV steroid to use is based on clinician preference.

**Anticholinergics**

Anticholinergic agents used in acute severe asthma are muscarinic antagonists that inhibit the action of acetylcholine at muscarinic receptors. They block airway smooth muscle contraction and secretion of mucus via parasympatholytic action, resulting in decreased intracellular cyclic guanosine monophosphate levels. The typical anticholinergic agent that is used in status asthmaticus is ipratropium bromide, given its action of bronchodilation with poor systemic absorption and its short-acting nature. Nebulized ipratropium bromide is given at 125–500 mcg/dose every 4–6 h in the PICU. Side effects of ipratropium bromide can include dry mouth, flushing, tachycardia, dizziness, mydriasis, and blurred vision due to accidental topical ocular exposure. Long-acting anticholinergic agents are used as controller medications for asthma management of outpatients, but do not have a role in status asthmaticus. There are not sufficient studies that report superiority of addition of ipratropium bromide to albuterol usage in the PICU in comparison to albuterol alone. There does seem to be some benefit in utilizing ipratropium bromide in the emergency department to prevent admission to the hospital, though the same effect has not been evaluated in the PICU [1,2,10,25,26].

**Methylxanthines**

Methylxanthine agents are another adjunctive class of medications used in the setting of status asthmaticus. Aminophylline is the methylxanthine commonly used in acute severe asthma, in which the active agent is theophylline. Theophylline is formed by the methylation of xanthine and is thought to act as a phosphodiesterase-4 inhibitor, thereby decreasing the degradation of cAMP, which then results in bronchodilation. It also has anti-inflammatory and immunomodulator actions and increases diaphragm contraction and respiratory drive. Another possible mechanism of action of methylxanthines is that they cause inhibition of adenosine receptors, leading to bronchodilation and decreased histamine production.

Aminophylline is typically administered as a loading IV bolus dose followed by a continuous IV infusion. The loading dose is 1.25 mg/kg/dose, followed by continuous infusion of 0.5–1 mg/kg/h depending on age. For patients with impaired cardiac or hepatic function, continuous infusion dose should be reduced to 0.25 mg/kg/h [1,2,10].

While enteral theophylline is available, it is typically not used given its narrow therapeutic index, which requires frequent monitoring, and its interaction with other drugs through the cytochrome P450 pathway [26].

Methylxanthines have significant side effects, due to which a narrow therapeutic window should be maintained. Adverse effects that can be seen are nausea, vomiting, seizures, tachycardia, arrhythmias, hypertension, tremors, and even death. Methylxanthines are also noted to be weak diuretics. It is important to monitor serum theophylline levels for any patient on an aminophylline infusion to prevent side effects. The goal serum level is 10–15 mcg/mL, and titrations should be performed to maintain those levels. Levels should be checked 12 h from the initiation of the drug or after every dose adjustment [1,2,10].

The use of methylxanthines has been noted to have a positive effect in improving lung function in acute severe asthma in conjunction with other medications such as albuterol and steroids. However, its use has not been correlated with a decreased hospital length of stay [27]. In a prospective study performed by Wheeler et al., theophylline was compared to terbutaline alone and the combination of theophylline and terbutaline (all patients also received IV methylprednisolone and continuous albuterol) in the setting of status asthmaticus. There was no significant difference in effectiveness between the three intervention groups, though, of note, hospital cost was significantly lower in the theophylline alone group [28].

**Magnesium**

Magnesium is another medication that is used in status asthmaticus. It prevents calcium uptake and, therefore, results in bronchodilation. It is available in both the nebulized and IV forms [1]. While nebulized magnesium, either alone or in combination with nebulized albuterol, seems to be well tolerated, it has not been proven to have consistent benefit in acute severe asthma and is, thus, not routinely recommended [29,30,31].

IV magnesium has been used both in the emergency room and ICU for pediatric status asthmaticus. However, there is not strong evidence suggesting that IV magnesium reduces length of hospital stay, due to limited studies published on this matter, and further studies need to be performed to clearly understand the benefits. A systematic review looking at IV magnesium for pediatric status asthmaticus in the emergency room found that its use profoundly decreased hospital admissions, but the number of studies that were able to be included were limited [32]. A single center randomized control study by Singhi et al. demonstrated that IV magnesium was more efficacious and safer than using IV terbutaline or aminophylline when added to inhaled β2 agonists and steroids [33]. IV magnesium can either be given as a bolus dose (25–75 mg/kg/dose over 20–60 min with a maximum of 2 g/dose) or as a continuous infusion.

For those receiving a continuous infusion, typical regimens that have been reported utilize a loading dose of 25–75 mg/kg/dose followed by infusions over about 4 h (40–50 mg/kg/h) or for >24 h (~18–25 mg/kg/h) [34,35]. In patients receiving a continuous infusion, serum magnesium levels are recommended to be monitored every 4 h, and a level of 4–6 mg/dL is often targeted. Various dosages have been used for both bolus doses as well as continuous infusion, though no comparative studies have proven one particular dose or regimen to be superior to others [34]. Side effects that can be seen include hypotension, nausea/vomiting, muscle weakness, flushing, and sedation. A systematic review noted that side effects were predominately seen in patients receiving magnesium for >24 h [34].

**Oxygen Therapy**

Due to intrapulmonary shunts from mucus plugging, atelectasis, and hyperinflation, patients in status asthmaticus can present with hypoxemia. There may also be some ventilation–perfusion mismatch due to albuterol nebulization, which can result in hypoxemia. Humidified oxygen can be utilized for such patients.

Due to severe bronchoconstriction, there can be turbulent airflow in the smaller airways. One way to allow for more laminar flow into the airways is through the use of heliox, a mixture of both helium and oxygen. Helium is a less dense gas, thereby reducing the Reynolds number and allowing for more laminar gas flow into the airways and decreasing the work of breathing in situations with obstructive airway lesions [1]. Heliox may result in improved delivery of nebulized treatments in patients with status asthmaticus with improved pulmonary function and decreased hospital stay in comparison to patients not receiving heliox therapy [36], though this benefit has not been consistently proven [37,38]. While the use of heliox for status asthmaticus has decreased over the last decade, it is noteworthy that there are negligible side effects, and it may be reasonable to have a trial of heliox as an adjunct to other therapies [26,38]. The duration and optimal mixture of heliox to be used for status asthmaticus has not been studied, though typical mixtures that are made to receive full benefit of the lower density of the gas are 80:20 or 70:30 helium/oxygen mixtures, so they cannot be used in patients with higher oxygen requirements [1].

**Noninvasive Ventilation**

Noninvasive ventilation, such as high flow nasal cannula (HFNC), continuous positive airway pressure (C-PAP), and bilevel positive airway pressure (BiPAP), can be trialed in status asthmaticus. Continuous positive airway pressure, as seen in HFNC and C-PAP, is thought to stent open small airways and allow better penetration of bronchodilators to small airways. BiPAP also gives positive inspiratory pressure, which may decrease accessory muscle usage and recruit collapsed alveoli to reduce atelectasis. It can also increase functional residual capacity and, thus, result in improved oxygenation and ventilation. More importantly, using noninvasive positive pressure can result in significantly decreased rates of invasive mechanical ventilation for status asthmaticus and, therefore, decrease morbidity and mortality due to asthma [39]. Overall, rates of noninvasive ventilation use have increased nationally, and its use has decreased invasive mechanical ventilation. There are, however, considerable differences in practical usage of noninvasive ventilation in various sites and, as such, no standardized approach in the determination of patients that qualify for noninvasive ventilation. This is determined mainly by clinical practices of practitioners [40,41]. Of note, most studies looking at noninvasive ventilation refer to the use of C-PAP and BiPAP. Studies looking at HFNC in pediatric status asthmaticus are limited, though some show that it is comparable to an aerosol mask in terms of PICU and hospital length of stay, though it was anecdotally better tolerated. Optimal flow rate to provide adequate delivery of medication is not known and is debated among providers. Studies have given various results with regards to albuterol delivery via HFNC at lower versus higher flow rates [42]. Further studies on HFNC are required to better assess its efficacy.

Some practical considerations to be noted with the use of noninvasive ventilation are concerns for patient tolerance and gastric distension. It is important to have well-fitting interfaces for positive airway pressure and patient cooperation, which may be difficult with an agitated child. Patients may potentially need sedation to allow for noninvasive ventilation use, a topic that will be addressed in a separate section. In a prospective randomized controlled trial examining noninvasive ventilation in status asthmaticus, gastric distension for the positive pressure was reduced by placing a nasogastric tube and aspirating air from it [43]. There have not been significant adverse events such as pneumothorax due to barotrauma reported secondary to noninvasive ventilation [39].

**Mechanical Ventilation**

Less than 1% of children with status asthmaticus require intubation. An analysis of the Virtual Pediatric Systems database from 2009 to 2019 showed that about 5% of patients admitted to the PICU with status asthmaticus required intubation and mechanical ventilation, with this percentage decreasing over time. Mortality rates in asthma are about 0.35% of PICU admissions due to asthma, though of those patients, about 98% were mechanically ventilated. Patients who were 6–12 years of age were at the highest risk of requiring invasive mechanical ventilation, as were male patients in comparison to female patients [44].

Given the significant adverse effects and mortality associated with mechanical ventilation in an asthmatic, all efforts are taken to prevent intubation. While there are not standardized indications for timing of intubation for status asthmaticus, clear indications would be cardiac or respiratory arrest, significant hypoxemia unresponsive to supplemental oxygen, obtundation, and respiratory failure [1,2].

Certain considerations need to be taken during the intubation of patients in status asthmaticus. It is important to note that these patients at the time of intubation are often hypoxemic, acidotic, and fatigued. Also, with hyperinflation of lungs seen in status asthmaticus, this can result in decreased preload to the heart and cause hypotension, especially if they were already on noninvasive positive pressure ventilation. Care must be taken to select medications for intubation that do not further exacerbate the aforementioned issues. Oxygen saturations should be optimized as much as possible before intubation and fluid status. While different drugs may be used for intubation, ketamine is often selected given its sedative, analgesic, and bronchodilatory properties, as well as the fact that due to its sympathetic stimulation, it does not cause hypotension or bradycardia. However, it may result in increased laryngeal secretions. A nondepolarizing neuromuscular agent, for example, rocuronium, is also often used during intubation.

Once the patient is intubated, it is important to understand that while mechanical ventilation will allow for inhaled gases to be pushed through edematous and mucus-filled airways, due to those same considerations, a prolonged expiratory phase will be needed. Failure to allow for full exhalation can result in distended terminal bronchi and alveoli (air trapping), which could lead to pneumothoraxes and barotrauma [14]. Similarly, high respiratory rates can result in hyperinflation and further hemodynamic instability. Thus, patients with status asthmaticus who are mechanically ventilated require low respiratory rates and prolonged expiratory times to ensure full exhalation. Some amount of hypercapnia is permissible to prevent high respiratory rates and high airway pressures that can result in hyperinflation.

Various modes of ventilation can be used to manage pediatric status asthmaticus, but an ideal mode of ventilation is not established. Using a pressure control mode can allow better control of mean airway pressures and peak inspiratory pressures, though it may have variable tidal volumes. With a volume control mode, while tidal volumes and minute ventilation can be controlled, airway pressures cannot be fully regulated. Volume control modes also allow for measurements of plateau pressures and the ability to compare peak-to-plateau pressures over time, which can indicate airway resistance and overall response to therapy. Neither mode has been proven to be superior in management, and full understanding of advantages and disadvantages of each mode should be present while treating these patients.

Due to small airway inflammation and increased airway resistance in asthma, the alveoli often fail to return to their functional residual capacity at the end of exhalation, which can lead to air trapping and hyperinflation. This is known as Auto PEEP or intrinsic PEEP. Auto PEEP can result in increased work of breathing, impaired ventilation, and can result in hemodynamic compromise and barotrauma. For patients who are mechanically ventilated, whether PEEP should be used or not is controversial. It is thought that setting a PEEP on the ventilator prevents dynamic collapse of the small airways at the end of exhalation and allows full exhalation and decreases the risk of Auto PEEP. On the other hand, there is a school of thought that the application of PEEP can result in worse hyperinflation given that there may not uniformly be dynamic collapse of the small airways in asthma, especially since the significant bronchoconstriction occurs in the larger airways. For patients spontaneously breathing, applying PEEP from the ventilator may help the patient better trigger the ventilator and improve synchronization. If PEEP is applied, it is typically set at a level below Auto PEEP. A total of 80% of the Auto PEEP level has been suggested as the level at which there is less risk of increasing hyperinflation or hemodynamic compromise, though for patients who are not spontaneously breathing, even a PEEP of 0 can be used, as extrinsic PEEP may not be needed given the patient’s Auto PEEP [1,45,46,47,48,49]. It is important to individualize the titration of PEEP in status asthmaticus to the patient’s physiology, especially given the heterogeneity of the disease. While titrating PEEP, plateau pressures should be monitored to see if the increase in PEEP is resulting in increased hyperinflation.

Given the significant challenges in ventilating patients with status asthmaticus, patients may be extubated, though there are still symptoms of asthma. The endotracheal tube itself can irritate the airway and result in bronchospasm; therefore, extubation should be targeted sooner rather than later [47,50]. Additionally, the endotracheal tube acts as an additional exhalation resistor in the setting of an existing obstructive pathology [51].

**Inhalational Anesthetics**

Certain inhalational anesthetics can also be used in the setting of intubated patients with status asthmaticus. Anesthetic drugs such as halothane, isoflurane, enflurane, and sevoflurane can be used for their bronchodilatory properties. These agents are significant smooth muscle relaxers and, thus, bronchodilators. While there are no studies comparing the efficacy of anesthetic drugs to other therapies used in asthma, case series have shown that the use of inhalational anesthetics can allow for improved ventilation. There are no studies to show that any of these agents are more superior than other anesthetic agents in the setting of status asthmaticus, though there are well-known risk profiles, such as hepatotoxicity in halothane. Based on prior reports, isoflurane seems to have been used the most in the pediatric age group. Isoflurane has a shorter onset of action and fewer cardiovascular or nephrotoxic effects.

Some side effects that have to be monitored for are hypotension, decreased renal perfusion, and cardiac arrhythmias. Given the intricacies of using an inhalational anesthetic, only those familiar with its use should be administering it and in charge of monitoring it. This often requires an anesthesiologist and specific ventilators that are capable of dispensing these drugs to be used for this purpose [1,46,52].

**High-frequency oscillator ventilation (HFOV)**

Typically, HFOV is not used in status asthmaticus due to the concerns for worsening hyperinflation from the higher pressures used in comparison to conventional mechanical ventilators. However, there have been case reports of pediatric patients where HFOV was used for status asthmaticus and was able to prevent the need for ECMO. The rationale behind its benefit is that the negative pressure created during expiration may improve ventilation and avoid air trapping and minimize changes in pressure in the small airways. This concept would also decrease atelectasis due to mucus and edema in the small airways. Typically, an “open airway strategy” is used with sufficiently high mean airway pressure (MAP) to stent the airways and lower frequencies. However, there must be close monitoring to prevent hemodynamic instability in the setting of HFOV and barotrauma. Further studies need to be performed to truly explore the efficacy of HFOV in status asthmaticus [53,54,55].

**Extracorporeal Membrane Oxygenation (ECMO)**

ECMO has been reported to be used in patients with status asthmaticus that has not been responsive to all other forms of management. As per the Extracorporeal Life Support Organization (ELSO), less than 4% of patients who have required ECMO for a respiratory cause have required it for status asthmaticus. The survival rate for patients requiring ECMO for an acute asthma exacerbation is >83% [1,46]. Indications for ECMO in these patients include significant hypoxemia despite maximal therapy and cardiorespiratory arrest. It can also be used in patients with significant hypercarbia despite high plateau pressures. Utilization of ECMO in those patients allows for less risk of barotrauma and hemodynamic instability. While placing a patient does not reverse the primary process that triggered the acute asthma exacerbation, it does allow the lungs to have decreased ventilator-associated lung injury while in “rest” settings and give time for the underlying process to reverse itself. However, it is important to note that placement on ECMO has its own set of possible complications, and ECMO is typically only reserved for patients who have failed other therapies [1,2,26,46].

Given that the issue in asthma is typically hypercarbia, there is a form of extracorporeal support known as pumpless extracorporeal lung assist. In this form, the patient’s own arterial pressure drives the blood flow through the gas-exchange membrane, resulting in the lack of need of a mechanical pump and, thus, decreased anticoagulation. However, this has been used in adult patients, not in pediatric patients [26,46].

**Sedation, Analgesia, and Neuromuscular Relaxation**

Various sedation, analgesia, and neuromuscular relaxants can be used in the setting of status asthmaticus, both for noninvasive and invasive mechanical ventilation. Dexmedetomidine can be used as an anxiolytic and can be used for both invasive and noninvasive mechanical ventilation. Its mechanism of action is via the inhibition of central sympathetic outflow by blocking alpha receptors in the brainstem, resulting in sedative and anxiolytic properties. There is growing research demonstrating the use of dexmedetomidine for patients on BiPAP/C-PAP to help improve patient compliance with the interface and, thus, improve its efficacy. It can also be used in invasive mechanical ventilation for sedation to prevent ventilator dyssynchrony. Its use is noted to be quite safe, though noted side effects are bradycardia and hypotension [56].

Ketamine is an anesthetic that has been favored in status asthmaticus due to its bronchodilatory properties. It is a dissociative anesthetic agent that is thought to work through the blockage of N-methyl-d-aspartate (NMDA) receptors. Its role in status asthmaticus has been postulated to be through the blockage of NMDA receptors in the airway, resulting in the release of norepinephrine and other catecholamines and, thus, bronchodilation. It can be given either intramuscularly, intravenously, or intranasally, though the intravenous route is most commonly used for the management of status asthmaticus. It is typically administered as a bolus dose of 1–2 mg/kg, followed by a continuous infusion of 0.75–2 mg/kg/h [1,2,26]. There have been limited studies showing the potential benefits of ketamine in status asthmaticus, possibly resulting in preventing the use of mechanical ventilation. However, this evidence is limited, and further studies need to be carried out [57,58]. Important side effects to be noted are sialorrhea and increased airway secretions, increased heart rate and cardiac output, and hallucinations. While it can be used in nonintubated patients, it is important that it is administered by practitioners that are familiar with its use and able to quickly intervene in case of its failure [1,2,26,58]. Ketamine is often a drug of choice in patients with status asthmaticus who are mechanically ventilated given its anesthetic and bronchodilatory effects.

Patients who are mechanically ventilated due to status asthmaticus may need neuromuscular blockade to prevent ventilator dyssynchrony that may worsen dynamic hyperinflation and also to have appropriate gas exchange. Neuromuscular blockade can also reduce the risk of sudden cough-induced pulmonary barotrauma. However, it is important to discontinue neuromuscular blockade when feasible, given the strong association of neuromuscular blockade agents with steroids resulting in myopathy. The duration of muscle weakness due to this myopathy can be varied and can even result in prolonged ventilator time, and neuromuscular blockade agents should be used judiciously. While the aforementioned myopathy is most often associated with the use of aminosteroid-based neuromuscular blockers, such as vecuronium and rocuronium, there are reports of incidences of muscle weakness with benzylisoquinolinium compounds, such as cisatracurium and atracurium [1,2,59].

## 4. Conclusions

Though there is a high incidence of asthma and significant morbidity and mortality associated with pediatric status asthmaticus, there are not standardized recommendations to guide management of these patients, especially in the pediatric ICU. There are multiple different adjunctive therapies available to providers, with medical management summarized in Table 2. However, there remains ample opportunity for further studies to be performed to allow for more well-defined guidelines and to prove the clear-cut efficacy of these therapies. Though there may not be clear benefits for all adjunctive therapies, it may be worthwhile to consider them while carefully weighing the risk profile. A limitation of this review article is that only peer-reviewed and PubMed indexed articles were included in this review, and, as such, there may be articles that did not meet these criteria and, thus, were not included. In conclusion, this review underscores the critical importance of effective management strategies tailored to pediatric patients with status asthmaticus in the ICU, highlighting the need for further research to refine and optimize current practices.

## Figures and Tables

**Table 1:** Pediatric asthma severity scoring systems.

Scoring System	Components	Scoring Thresholds	Additional Comments
Pediatric Asthma Score (PAS) [4]	Respiratory rate Breath sounds/dyspnea Wheezing Accessory muscle use Oxygen measurement	Mild asthma: ≤7 Moderate asthma: 8–11 Severe asthma: ≥12	Only to be used in patients ≥ 2 years of age
Pediatric Respiratory Assessment Measure (PRAM) [5]	Oxygen saturations Suprasternal retractions Scalene muscle contraction Air entry Wheezing	Mild: 0–3 Moderate: 4–7 Severe: 8–12	Originally developed for age 3–6 years, but validated for 1–17 years of age
Clinical Respiratory Score (CRS) [6]	Respiratory rate Auscultation Use of accessory muscles Mental status Room air SpO_2_ Color	Mild: ≤3 Moderate: 4–7 Severe: 8–12	

**Table 2:** Summary of medical management for status asthmaticus.

Medication Category	Mechanism of Action	Name	Available Routes	Dosage	Adverse Effects
Beta Agonist	Stimulation of β adrenergic receptors-> bronchodilation and inhibit mast cell mediator release.	Albuterol	Inhaled (MDI or nebulized), oral (not recommended), IV (outside of the US).	Intermittent inhaled: 0.05–0.15 mg/kg/dose. Continuous inhaled: 0.15–0.5 mg/kg/h (max 10–20 mg/h).	Tachycardia, lower MAP and DBP, hypokalemia, tremors.
		Epinephrine	SC, IV, IM, Inhaled	0.01 mg/kg/dose (max 0.5 mg)	Tachycardias, arrhythmias
		Terbutaline	MDI (Outside of the US), oral, SC, IV.	SC: 0.1 mg/kg/dose (max 0.3 mg). IV: 10 mcg/kg loading dose followed by 0.1–0.5 mcg/kg/min infusion.	Tremors, skeletal muscle twitches, hypokalemia, tachycardia, hypotension, increased cardiac enzymes, ventricular tachyarrhythmias.
Steroids	Decrease airway inflammation.	Prednisone/Prednisolone	PO	1–2 mg/kg/day divided 1–2 times per day.	Hypertension, anxiety, irritability, gastritis, hyperglycemia.
		Methylprednisone	IV	0.5–1 mg/kg/dose Q6H (max 60 mg/dose).	
		Dexamethasone	IV, IM	0.6–1 mg/kg/day Q6H.	
		Hydrocortisone	IV	1.5–4 mg/kg/dose Q6–8 h.	
Anticholinergics	Muscarinic antagonists.	Ipratropium bromide	Nebulized	125–500 mcg/dose Q4–6 h.	Dry mouth, flushing, tachycardia, dizziness, mydriasis, blurry vision.
Methylxanthines	Phosphodiesterase-4 inhibitor.	Aminophylline	IV	1.25 mg/kg/dose (loading), followed by 0.5–1 mg/kg/h infusion.	Narrow therapeutic index—Levels to be monitored. Nausea, vomiting, seizures, tachycardia, arrhythmias, hypertension, tremors, death.
		Theophylline	PO, IV	PO (not used often) 5 mg/kg/dose. IV 4.6 mg/kg/dose (loading), followed by 0.4–0.8 mg/kg/h (based on age).	
Magnesium	Prevents calcium uptake-> bronchodilation.		Inhaled, IV	Inhaled: Not recommended routinely. IV intermittent: 25–75 mg/kg/dose (max 2 g/dose). IV continuous: 25–75 mg/kg/dose loading followed by 40–50 mg/kg/h over 4 h or 18–25 mg/kg/h over >24 h.	Hypotension, nausea, vomiting, muscle weakness, flushing, sedation.

Summarized from multiple sources [1,2,10,14,16,17,22,23,24,25,26,34,35]. MDI: metered dose inhaler; IV: intravenously; US: United States; mg: milligram; kg: kilogram; h: hour; SC: subcutaneously; IM: intramuscularly; mcg: microgram; min: minute.

## Data Availability

The original contributions presented in the study are included in the article, further inquiries can be directed to the corresponding author.
